# The Experience of Stigma Within the Young Onset Dementia (YOD) Population: A Systematic Review of Qualitative Research

**DOI:** 10.1111/hex.70773

**Published:** 2026-07-21

**Authors:** Fiona Lowe, Kerry Hanna, Sarah Evans, Naheed Tahir, Clarissa Giebel

**Affiliations:** ^1^ Department of Primary Care and Mental Health University of Liverpool Liverpool UK; ^2^ School of Allied Health Professions and Nursing University of Liverpool UK; ^3^ NIHR Applied Research Collaboration North West Coast Liverpool UK

**Keywords:** discrimination, marginalisation, social isolation, stigma, young onset dementia

## Abstract

**Introduction:**

People with Young Onset Dementia (YOD) are recognised as having a higher risk of being marginalised within society and in the care and support they receive. The consequences of stigma on people with YOD must be better understood to help reduce experiences of discrimination and increase awareness for this population. This qualitative systematic review aimed to synthesis the data on the experience of stigma within the YOD population globally.

**Methods:**

Five electronic databases (Scopus, MEDLINE, PubMed, Web of Science, and PsycINFO) were searched in December 2025. The quality of the studies was assessed using the Critical Appraisal Skills Programme Tool. Data were analysed using thematic synthesis. One researcher assessed the title and abstracts of the articles retrieved against the inclusion criteria. Included articles were then read in full by the same researcher. A second researcher reviewed 10% of these articles at both stages.

**Results:**

2994 articles were screened, and 17 studies were included in this review. Four themes were identified: Public perception and beliefs about YOD, Structural stigma and barriers to service access, The role of stigma in social isolation, and Overcoming stigma.

**Discussion:**

This review highlights the pervasive and multifaceted nature of YOD, offering insights into individual and service level strategies needed to mitigate the negative consequences of stigma. The findings highlight the need for specialist age‐appropriate support services alongside public and professional education on YOD to address misconceptions about dementia in younger adults. Community and government‐led initiatives are recommended to help reduce negative stereotypes, social isolation and improve access to early diagnosis and tailored support for our YOD communities.

**Patient and Public Involvement:**

Not applicable.

## 1 Introduction

Stigma is defined as the presence of negative stereotypical thoughts, feelings, behaviours and beliefs [1]. Dementia stigma is a major concern within the dementia community and advocacy services [1]. People with Young Onset Dementia (YOD) are recognised as having a higher risk of being stigmatised within society and in the care and support they receive [2]. The term cultural stigma refers to shared negative beliefs, prejudices and discriminatory systems upheld within society [1]. Courtesy stigma refers to the stigma experienced by family, friends and carers due to their association with a person living with a condition and their role in caring for them [3, 4]. Erving Goffman developed a modern conceptualisation of stigma describing it as when a person is seen as tainted by society and disqualified from social acceptance because of a specific characteristic [5]. More recently, Link and Phelan [6] developed a framework to understand stigma using six concepts. These models conclude that when stigma occurs at the individual, societal or institutional level, it isolates and excludes people from participating in society [5].

Media and societal portrayals of dementia reflect the dominance of older people with a diagnosis and suggest that individuals inevitably have severe impairments and reduced quality of life [7]. This narrative can reinforce the cultural stigma and negative image around dementia. It exacerbates and influences society's negative narratives about receiving a dementia diagnosis and living with the condition [8]. Research suggests that dementia stigma can delay help‐seeking behaviours and access to a diagnosis due to the public's fear of dementia and the reluctance from healthcare professionals to give a diagnosis [9]. The literature suggests that experiencing stigma can impact negatively on the quality of life of those living with dementia and their family members [10]. Stigma can also create several barriers to accessing necessary post diagnostic care and support for those who require it within the dementia community [11, 12]. Research on dementia stigma has shown that it may lead to experiences of discrimination within healthcare services, and individuals reporting that they feel ignored, dismissed, and denied appropriate care provisions [13]. Furthermore, findings indicate that perceived stigma by those living with a dementia diagnosis is associated with poorer mental health outcomes, lower self‐esteem and reduced engagement in activities [14].

Young Onset Dementia (YOD) is a term used to describe people who experience the onset of dementia symptoms before the age of 65 years or receive a dementia diagnosis before the same age [15]. It is estimated that 3.9 million people are diagnosed with YOD globally, of whom 70,800 live in the UK [16]. Research investigating the experiences of individuals living with YOD and their carers has highlighted the unique challenges faced by this population, including barriers to accessing age‐appropriate care [17, 18, 19]. Existing research illustrates the negative psychosocial impact of receiving a diagnosis at an earlier stage in life and the need for specialist YOD community and residential services, that is currently limited globally [20]. Factors that contribute to the risk of stigma and marginalisation for people living with YOD include challenges reported in the detection of the condition, misdiagnosis and confusion around the symptoms and presentations in YOD [21].

Research suggests that feelings of shame associated with a YOD diagnosis can result in initial denial of the condition by those affected, and by health and social care providers [2]. This can result in limited access to helpful information on available resources and support to those impacted by YOD [22]. This is further evidenced in the reduction in access to appropriate treatment options and post‐diagnostic care to this cohort of younger adults who are living within a society that perceives dementia as an older adult condition [22]. It is vital that increased knowledge and information about the consequences of stigma and discrimination among people with YOD is provided. Furthermore, the specific barriers that negative stereotypes can have on this population should be examined and synthesised to understand the psychosocial implications of the condition and the strategies needed to reduce it [2].

Existing research exploring the lived experiences of people with YOD and their families have identified stigma within the findings. Spreadbury and Kipps [23] conducted a systematic review on YOD and found that social isolation, misunderstanding and marginalisation was experienced widely within the YOD community. A review exploring YOD and self‐identity has emphasised the psychosocial burden and societal responses that contribute to stigma [24]. Within these reviews, stigma is typically reported within a theme and not the primary focus of the analysis. There does not appear to be any systematic review analysing the literature on the experience of stigma in the YOD population specifically. Therefore, the aim of this systematic review was to explore and synthesise the global evidence base on the experience of stigma surrounding a diagnosis of YOD. This review aims to understand how stigma influences people with YOD and families accessing a diagnosis and subsequent dementia care. It aims to identify potential stigma‐related barriers to accessing support for YOD populations. Lastly, this review aims to understand the psychosocial and emotional implications of stigma on the YOD population.

## 2 Methods

### 2.1 Protocol and Registration

This qualitative systematic review was registered on PROSPERO [ID: CRD42023452779] prior to any searches undertaken. This review followed the Preferred Reporting Items for Systematic Reviews and Meta‐Analyses (PRISMA) guidelines [25].

### 2.2 Search Strategy

The primary researcher conducted a systematic search across five major databases: Scopus, MEDLINE, PubMed, Web of Science, and PsycINFO. The search strategy was developed using the PICO framework (Patient/Participant/Population; Intervention/Exposure; Comparison; Outcome [26]). Search terms for the population included ‘young onset dementia’, ‘early onset dementia’, ‘presenile dementia’, and ‘younger onset dementia’. The search terms related to exposure included ‘stigma’, ‘discrimination’, ‘bias’, ‘attitude’, ‘marginalisation’, ‘prejudice’, and ‘exclusion’. There were no restricted search terms applied to the comparison or outcome categories. The basic search syntax was entered into each database as follows: ((young onset dementia) OR (early onset dementia) OR (presenile dementia) OR (younger onset dementia)) AND ((Stigma*) OR (Discriminat*) OR (Prejudic*) OR (Marginal*) OR (Bias*) OR (Attitude*) OR (Exclu*)).

The search strategy was limited to peer‐reviewed journal articles published in English or German between 2010 and 2025. The initial search was conducted in April 2024, followed by updated searches in February 2025 and December 2025. The citations were imported into an online systematic review software platform, Rayyan [27]. Following the removal of all duplicates, the titles, abstracts, and full‐length texts were screened by the primary researcher in successive stages against the inclusion and exclusion criteria. The Rayyan account was shared with the research team for cross‐checking. Lastly, hand searches of the reference lists of included studies was conducted.

### 2.3 Inclusion and Exclusion Criteria

This systematic review included qualitative, empirically based journal articles that examined the experience of stigma and other related concepts (e.g. discrimination, marginalisation, social exclusion and bias) among people with YOD, families, carers and professionals working in this area. Eligible studies were published in English or German, available as full‐length articles, and included participants aged 18 years or older. Studies that explored the experience of YOD populations only were eligible. Studies where the person with YOD had received a diagnosis of dementia before 65 were eligible.

To ensure consistency in article selection, publications were excluded if they did not report findings on the experience of stigma towards people with YOD. Articles were excluded if participants were diagnosed with dementia after 65 years of age. Articles including all age dementia populations were excluded to ensure the analysis focused on stigma within YOD specifically, as combining younger and older populations risks diluting the unique psychosocial and life stage related experiences of people with YOD.

Articles were excluded if they did not focus on human participants, were systematic reviews, conferences, discussion papers or dissertations and thesis. Lastly, articles that were unavailable at full text in English or German and papers with participants under 18 years of age were excluded.

### 2.4 Study Selection

The titles and abstracts of the articles retrieved from the five databases were screened against the inclusion criteria by the primary researcher. Articles were excluded if they failed to meet the inclusion criteria at stage one. The remaining articles were read in full at stage two and 17 studies were included in the final review. A second researcher independently reviewed 10% of the articles at both stages. Any discrepancies at both stages were resolved through discussions with members of the research team.

### 2.5 Assessment of Risk of Bias

A quality assessment was completed using the Critical Appraisal Skills Programme [28] checklist. The CASP tool is used to appraise the strengths and limitations of qualitative research methodologies. The tool comprises of 10 questions that assess the quality of the methodologies used, the appropriateness of the method and research design and whether the findings have meaningful implications for the population being assessed [28]. The CASP tool is routinely used within health and social care related studies [29]. It is endorsed by the Cochrane Qualitative and Implementation Methods Group and the World Health Organisation for use in qualitative synthesis [29]. It was deemed the most appropriate tool for the context of this review by the research team. In line with the Centre for Reviews and Dissemination guidance, studies were included even when risk of bias was indicated [30]. Although appraisal scores did not influence study selection, the CASP scores were considered during the interpretation of the findings.

### 2.6 Data Extraction

Data were extracted from 17 studies included in this systematic review. FL examined each study in full to identify information relevant to the aims of the review. A Microsoft excel spreadsheet was developed to organise the extracted data, including author, title, year of publication, study location, sample size, participant demographics (e.g. role, gender, age range, age at diagnosis of YOD, ethnicity), study methodology, data analysis and study design where specified.

### 2.7 Data Synthesis

The research team discussed the findings from the 17 studies included in the review and collaboratively generated the final themes. Data were synthesised using thematic synthesis [31]. In the first stage of thematic synthesis, data familiarisation, FL examined and extracted contextual and participant data from each study. Next, the studies were imported into NVivo, where FL coded the findings to identify patterns across the studies. Initial codes were developed and reviewed by the research team. This process led to the generation of descriptive themes [31]. In the final stage of synthesis, FL developed analytical themes by moving beyond the collective findings of the included studies. The final themes were refined by the research team to ensure rigour and consistency.

To enhance transparency and enable replication of the analytic process, an audit trail was developed to illustrate how descriptive findings moved to higher order analytical themes. For example, an initial descriptive theme ‘social withdrawal and avoidance’ identified across studies related to withdrawal from social networks, avoidance of social situations and reduced participation in community activities for people with YOD and families. These experiences were linked to fear of judgement, embarrassment, and anticipated stigma. This led to the development of the analytical theme ‘The role of stigma in social isolation’, situating social withdrawal and avoidance within broader stigma processes. Another initial descriptive theme described strategies used by people with YOD and their families to manage stigma. At a descriptive level, these findings were grouped into ‘coping with stigma’ and ‘responses to stigma’. Next, these findings were developed into the analytical theme ‘Overcoming stigma’ to capture both individual coping strategies and broader efforts to challenge and reduce stigma at interpersonal and societal levels.

### 2.8 Research Team

The primary researcher, FL identified as a white female trainee clinical psychologist. They have personal experience of being an unpaid carer. The primary and secondary research supervisors (CG and KH respectively) are white, female, academic researchers with a wealth of qualitative research experience in the field of dementia and health inequalities. KH is a clinician working with patients with dementia. The second reviewer was a white female trainee clinical psychologist. This reviewer contributed to the screening of studies, as well as cross checking data extraction and quality appraisal. One public advisor was involved throughout the review process. They were an unpaid carer for an individual with YOD, from an ethnic minority background. This research team was involved in the iterative process of the data analysis and theme development through regular team meetings and review of the included articles and data extracted. Decision making processes and reflections that developed during research meetings were recorded to promote credibility, transparency and trustworthiness within the review.

## 3 Results

**Figure 1:**
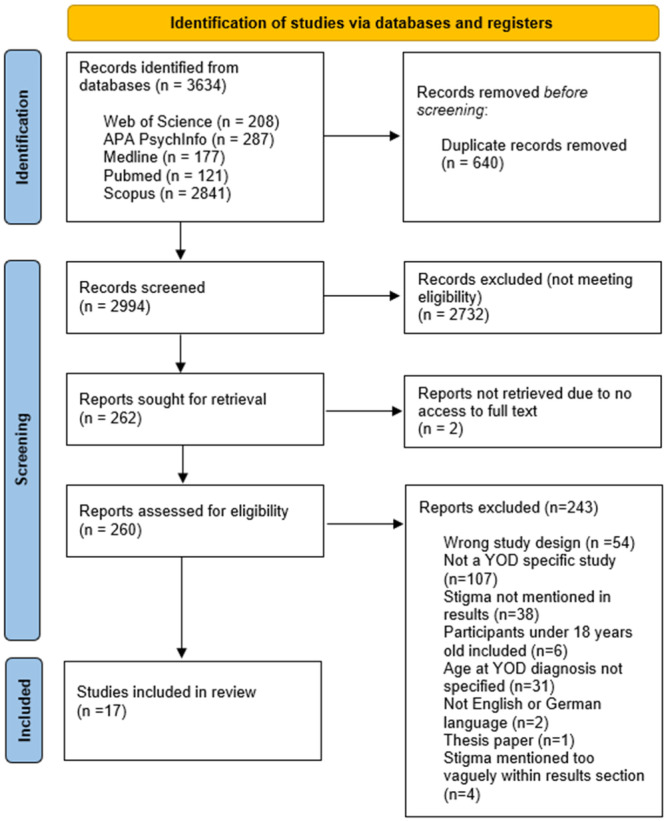
PRISMA flowchart.

A total of 3,634 articles were identified through database searches, resulting in 2,994 citations being screened at title and abstract level. 260 articles were read for inclusion/exclusion, with 17 articles included. The PRISMA flowchart (Figure 1) lists the reasons for full text article exclusion.

### 3.1 Characteristics of Included Articles

Table 1 provides an overview of the characteristics of the 17 studies included in this systematic review. The studies collectively explored the perspectives of three participant groups; people diagnosed with YOD before the age of 65 (*n* = 9), unpaid carers or family members (*n* = 8) and health and social care professionals working directly with YOD populations (*n* = 3). The professionals represented a range of health and social care roles, including occupational therapists, psychiatrists, nurses, geriatricians, social workers and psychologists. The sample sizes varied considerably across the studies, ranging from four participants to 31. Gender across the studies were mixed, although some samples were predominantly female, particularly among unpaid carers and professionals. Five studies reported ethnicity demographics. Three of these studies had participants that identified as exclusively White British. The studies were conducted across 12 countries, including Australia (*n* = 1), Belgium (*n* = 1), Canada (*n* = 1), China (*n* = 1), Finland (*n* = 1), Hong Kong (*n* = 1), Italy (*n* = 1), Ireland (*n* = 1), Israel (*n* = 1), Norway (*n* = 1), The Netherlands (*n* = 1), and the United Kingdom (*n* = 5). All 17 studies used qualitative methodological approaches employing designs such as interpretative phenomenological analysis, thematic analysis, grounded theory, and narrative analysis. The studies primarily used semi structured interviewing for data collection and/or focus groups.

**Table 1:** Study and participant characteristics.

Author and publication date	Study aim/research question	Country	Sample size	Participant characteristics (role, gender, age range, ethnicity, age at diagnosis)	Study design	Qualitative analysis	Method of data collection andduration of interviews
[32]	To explore family carers' perspectives on caregiving and living with people with YOD.	Ireland	9 Primary caregivers	Caregivers: 2 males, 7 females Age not reported People with YOD: Age range at time of diagnosis: 45–63 years Ethnicity not reported	Qualitative	Inductive thematic analysis	Semi structured interviews 30–60 min
[33]	To explore professionals' perspectives on the system and policy level factors influencing diagnostic journeys for YOD populations.	Australia	11 Healthcare professionals	Professional role: Occupational therapist, Psychiatrist, Nurse practitioner, Geriatrician, Enroled nurse, Neurology registrar, General Practitioner, Social worker, Psychologist 6 males, 5 females 21–51+ years Ethnicity not reported	Qualitative	Grounded theory constant comparative analysis	Semi structured in depth interviews 30–60 min
[34]	To explore the experiences of spousal caregivers for people with YOD to inform the development of efficient dementia services.	China	11 Spousal caregivers	Caregivers: 7 males, 4 females 56–63 years People with YOD: 55–63 years Ethnicity: Chinese	Qualitative	Content analysis using inductive and deductive approaches	Semi structured interviews 35–126 min
[35]	To explore the lived experience of spousal caregivers and consider the resources required for the YOD population.	Canada	12 Spouses or family caregivers	Caregivers: 4 males, 8 females Mean age: 55 years People with YOD: All diagnosed before 65 years Ethnicity not reported	Qualitative	Interpretative Phenomenological Analysis	Semi structured interviews Approx. 90 min
[36]	To explore how people influence their own lives and factors that impact positively and negatively on this process in YOD populations.	Finland	12 People with YOD	4 males, 8 females 54–65 years Ethnicity not reported	Qualitative	Theory guided content analysis using inductive and deductive approaches	Three focus groups 56–90 min
**Johnannessen et al., 2014**	To examine metaphorical expressions of everyday life experiences in YOD populations.	Norway	20 People with YOD	12 males, 8 females 54–67 years Diagnosed with YOD from 4 months to 3 years prior to participating Ethnicity not reported	Qualitative	Analysis of metaphors and comparison between original analysis	Interviews from previous study
[37]	To explore how people with YOD navigate disclosure of their diagnosis and understand how self‐disclosure changes.	United Kingdom	9 People with YOD	3 males, 6 females Mean age: 63.7 years Years since diagnosed: 1–10 years Ethnicity: White British	Qualitative	Narrative analysis	Semi structured interviews 25–62 min
[38]	To examine carers experience of spousal relationships and coping mechanisms in YOD populations.	United Kingdom	6 Primary caregivers	Caregivers: 3 males, 3 females 52–70 years People with YOD: Age range at time of diagnosis: 53–61 years Ethnicity: White British	Qualitative	Interpretative Phenomenological Analysis	Semi structured interviews 50–90 min
[39]	To explore service delivery for YOD including the challenges, barriers and facilitators identified by professionals.	Italy	31 Professionals	Professional role: Geriatricians (*n* = 2), Neurologists (*n* = 2), Psychiatrist (*n* = 1), Physician (*n* = 1), Music therapist (*n* = 1), Nursing assistant (*n* = 1), Nurses (*n* = 2), Psychologist (*n* = 14, Service manager (*n* = 1), Social workers (*n* = 3), Volunteers (*n* = 3) 4 males, 27 females 29–74 years Ethnicity not reported	Qualitative	Grounded methodology content analysis	Semi structured open‐ended interviews
[40]	To investigate the experience of caregiving and provide guidelines for effective service delivery in YOD populations.	Hong Kong	6 Spousal caregivers	3 males, 3 females 61–73 years People with YOD:Age range at time of diagnosis: 52–63 years Ethnicity: Chinese	Qualitative	Content analysis	Open ended interviews 55–128 min
[41]	To explore the experience of hope in YOD populations.	United Kingdom	6 People with YOD	4 males, 2 females 54–64 years Ambiguity of ethnicity in participant sample	Qualitative	Voice Centred Relational Method	Semi structured interviews Up to 60 min
[20]	To explore the experiences of people with YOD to understand the challenges they encounter and identifying areas for service development.	United Kingdom	14 People with YOD	Age range at time of interview: 57–67 years Age range at time of YOD diagnosis: 52–64 years Gender not reported Ethnicity not reported	Qualitative	Interpretative Phenomenological Analysis	Interviews 30–120 min
[42]	To examine the concept of personhood in YOD populations.	Canada	4 People with YOD	4 males 46–62 years Ethnicity not reported	Qualitative	Interpretative Phenomenological Analysis	Interviews
[43]	To examine the experiences, work values, and care needs of YOD populations.	The Netherlands	15 Individual interviews: 6 People with YOD and 9 Family members 4 Focus groups with 16 participants: 10 People with YOD and 6 Family members	**Individual interviews:** People with YOD: 5 males, 1 female 55–65 years Family members: 6 males, 3 females Age not reported	Qualitative	Thematic analysis	Interviews 45–90 min Focus group discussion 90–120 min
				**Focus groups:** People with YOD: 5 males, 5 females 54–65* Family members:6 malesAge not reported*Age not reported for 3 people. People with YOD diagnosed before 65 years Ethnicity not reported			
[44]	To explore why people with YOD use social media and what issues they encounter with this.	United Kingdom	11 People with YOD	8 males, 3 females 48–66 years Age range at time of YOD diagnosis: 45–63 years Ethnicity: White British	Qualitative	Thematic analysis	Semi structured interviews 35–80 min
[45]	To investigate families' perspectives on advance care planning for YOD populations.	Belgium	20 Dyads of 10 People with YOD and 10 Family caregivers	People with YOD: 8 males, 2 females Mean age at time of YOD diagnosis: 60 years Mean age at time of interview: 63 years Caregivers: 2 males, 8 females Mean age at time of interview: 60 years Ethnicity not reported	Qualitative	Constant comparative analysis	Semi structured in depth interviews
[4]	To explore the stigma formation process experienced by family caregivers and professionals who develop, manage and provide services to people with YOD.	Israel	6 Caregivers and 10 Professionals	Caregivers: 6 females 51–69 years People with YOD: Age range at time of diagnosis: 53–63 years Professionals: Role: Service providers (*n* = 4), Management staff (*n* = 6) 10 females 33–70 years Ethnicity not reported	Qualitative	Thematic analysis	Focus groups using semi structured interview guide

### 3.2 Assessment of Risk of Bias

The results of the quality appraisal assessment using the CASP tool are depicted in Table 2. The results indicated that there was some risk of bias, with few studies meeting all the requirements outlined in the CASP tool [28]. All studies outlined the aims of the research and used appropriate methodological approaches. All studies met the criteria for the appropriateness of the recruitment strategy. Similarly, all studies collected data that sufficiently addressed the aims of the research and to answer the research questions set out. All studies had a clear statement of findings. The quality appraisal tool identified limitations across several studies. Thirteen studies provided insufficient information on the relationship between the researchers and participants and did not critically reflect on the researcher's role, positionality and the risk of bias this may have caused in the interpretation of findings ([34, 35, 36]; Johannessen et al., 2024 [4, 20, 37, 39, 40, 41, 43, 44, 45]). Two studies did not sufficiently report whether ethical issues had been considered [20, 38]. The studies included were peer‐reviewed.

**Table 2:** Risk of bias assessment scores using the CASP Tool (CASP, 2018).

Author(s) and Publication Year	Was there a clear statement of aims of the research?	Is a qualitative methodology appropriate?	Was the research design appropriate to address the aims of the research?	Was the recruitment strategy appropriate to the aims of the research?	Was the data collected in a way that addressed the research issue?	Has the relationship between researcher and participants been adequately considered?	Have ethical issues been taken into consideration?	Was the data analysis sufficiently rigorous?	Is there a clear statement of findings?	Is the findings valuable?
[32]	Yes	Yes	Yes	Yes	Yes	Yes	Yes	Yes	Yes	Yes
[33]	Yes	Yes	Yes	Yes	Yes	Yes	Yes	Yes	Yes	Yes
[34]	Yes	Yes	Yes	Yes	yes	Can't tell	Yes	Yes	Yes	Yes
[35]	Yes	Yes	Yes	Yes	Yes	Can't tell	Yes	Yes	Yes	Yes
[36]	Yes	Yes	Yes	Yes	Yes	Can't tell	Yes	Yes	Yes	Yes
Johnannessen et al., 2014	Yes	Yes	Yes	Yes	Yes	Can't tell	Yes	Yes	Yes	Can't tell
[37]	Yes	Yes	Yes	Yes	Yes	Can't tell	Yes	Yes	Yes	Yes
[38]	Yes	Yes	Yes	Yes	Yes	Yes	Can't tell	Yes	Yes	Yes
[39]	Yes	Yes	Yes	Yes	Yes	Can't tell	Yes	Yes	Yes	Yes
[40]	Yes	Yes	Yes	Yes	Yes	Can't tell	Yes	Yes	Yes	Yes
[41]	Yes	Yes	Yes	Yes	Yes	Can't tell	Yes	Yes	Yes	Yes
[20]	Yes	Yes	Yes	Yes	Yes	Can't tell	Can't tell	Yes	Yes	Yes
[42]	Yes	Yes	Yes	Yes	Yes	Yes	Yes	Yes	Yes	Yes
[43]	Yes	Yes	Yes	Yes	Yes	Can't tell	Yes	Yes	Yes	Yes
[44]	Yes	Yes	Yes	Yes	Yes	Can't tell	Yes	Yes	Yes	Yes
[45]	Yes	Yes	Yes	Yes	Yes	Can't tell	Yes	Yes	Yes	Yes
[4]	Yes	Yes	Yes	Yes	Yes	Can't tell	Yes	Yes	Yes	Yes

### 3.3 Thematic Synthesis

Four overarching themes were developed to conceptualise the experience of stigma in YOD: (1) Public perception and beliefs about YOD; (2) Structural stigma and barriers to service access; (3) The role of stigma in social isolation; and (4) Overcoming stigma. Each theme is outlined below, with direct quotations from the articles included.

#### 3.3.1 Public Perception and Beliefs about YOD

Several studies outlined how negative public perception and cultural beliefs about a dementia diagnosis contributed to the experience of stigma within the YOD population [4, 20, 32, 34, 36, 37, 38, 39, 40, 41, 42, 44, 45, 46].

Social stigma and negative attitudes towards people with YOD arose from a lack of public awareness and education in communities ([32, 34, 38, 39]; Sakamoto et al., 2012). For example, participants reported that dementia is stereotyped as an older adult disease within society [32, 34, 37, 41]. Furthermore, negative beliefs and narratives such as dementia being *‘infectious’* promoted fear and shame among those affected [39]. Participants described a loss of social status, decreased self‐worth, and low self‐esteem due to limited awareness and understanding of YOD in society [38, 42, 45].

There was so little publicity about YOD; people don't understand, and we face discrimination everywhere in our daily lives.

(Caregiver [34])

I realise that there is a lot of confusion in the community, and fear too. Many people are scared as soon as they hear the term Alzheimer's. We should create a culture of education, starting with primary and elementary schools, to try to spread a culture of acceptance […]. Dementia is not infectious. We must not treat them as lepers, they are unfortunate people, but they have intact feelings.

(Professional [39])

Authors described how people with YOD and their caregivers perceived others in society as dismissive of their experiences, or as doubting the credibility of the diagnosis [32, 37, 38, 41]. Caregivers in one study described how there would be a mismatch between the physical presentation of the person with YOD compared to their cognitive and social decline (Lockeridge & Simpson, 2012). A lack of symptoms relating to memory exacerbated people's disbelief and lack of acceptance of the diagnosis [37]. This led to caregivers feeling undervalued in their roles and responsibilities because people overestimated the ability of the person with YOD, specifically when they were in shops or out for meals [38].

A few people said to me, ‘If I were you, I'd get that checked, I'd get a second opinion, I don't think he has dementia.’ They just couldn't believe it, he's so healthy.

(Wife [32])

[They] would say, ‘Oh, but you're fine… You don't look like you've got dementia’. And now I find these things quite irritating because what does someone with dementia look like?

(Female living with YOD [37])

For some people with YOD who were effectively masking their symptoms in the community, it appeared that they did not require support from caregivers. However, both carers and people with YOD described continually developing coping strategies to manage behaviours in public and avoid social embarrassment. These strategies included reducing choices for the person with YOD, withdrawing from social activities and avoiding public places [38]. The effort needed to present a competent image in society and the resulting public reaction resulted in participants feeling frustrated and misunderstood. This further reinforced self‐stigma and difficulties in accepting and coming to terms with the YOD diagnosis.

so I find I feel exhausted when I come away from there because I try so, so hard not to do something silly or let the side down… You sort of play acting in a way. Covering it, which of course compounds the problem, because then they think you're fine. You become your own victim of your own successful acting skills.

(Female living with YOD [37])

You know you sort of live a double life almost. You know, you're sort of, ‘yeah I'm fine, I'm fine.’ But underneath you're thinking well am I going to see my grandkids grow up?

(Person living with YOD [20])

Some participants described experiencing a lack of empathy and negative comments from the public due to their reaction to the symptoms of YOD [4, 32, 37, 44, 46]. Participants described instances where they became the target of discrimination when friends and wider networks began to treat them differently or began to withdraw from them due to feelings of fear and embarrassment about the condition [4, 42, 45]. These experiences of stigma reportedly progressed as the symptoms became more prominent.

When you have…dementia, people seem to treat you differently…. It's sad that you can be talking to somebody and once you mention the d‐word—dementia— you can tell the conversation is going to end or change.

(Male living with YOD [42])

He [her husband] is deeply offended by the fact that his friends have stopped calling and visiting him. I think [it's because] they are afraid of his odd behaviour.

(Wife [4])

It was discussed that cultural beliefs influenced the experience of stigma within YOD communities [34, 40]. Cui et al. [34] spoke about family members concealing the dementia diagnosis due to fears about genetic associated stigma on career progression and marriage. Pang and Lee [40] reported that dementia was a stigmatised disease within Chinese communities with a *‘loss of face’* felt by family members, especially when there was a diagnosis received at a young age.

We [my husband and I] felt that we were being stigmatised by others [our friends and neighbours]. For example, one of our neighbours always asked him: ‘Who is she [caregiver]?’ My husband replied: ‘She is my mom’. Then, he [the neighbour] laughed. I felt embarrassed…It's a ‘loss of face’ to have a husband with dementia, especially when he is so young. I will not seek help from others because they will look down on me. I felt inferior to others.

(Wife [40])

The lack of representation of dementia in younger populations led people with YOD to conceal their diagnosis from their wider social networks to try and fit in or strive to maintain their image and social status within society [20, 36, 37, 38].

I don't want it to colour their opinion of me anymore, because it is a stigma attached to dementia and I found that through the church members, not my close friends, but leaders, church members. And I don't want to affect other people's opinion of me before they get to know me. Yeah, get to know me, see what I'm really like and then I might tell them.

(Female living with YOD [37])

Overall, the three participant groups included in this review reported on the psychological and emotional impact of negative perceptions and stigmatising beliefs on the YOD population [38, 40, 42, 44, 45]. Professionals emphasised the need for increased education on YOD among younger populations and prioritisation of public health campaigns nationally to reduce the experiences of self‐stigma and social stigma (Ottonboni et al., 2021).

#### 3.3.2 Structural Stigma and Barriers to Service Access

A common theme within the included studies was the presence of stigma within health care and social care systems, which contributed to barriers in accessing a dementia diagnosis, specialist age‐appropriate services and adequate financial support [4, 33, 34, 37, 38, 43]. This was often reflected in a lack of awareness and understanding of YOD among health and social care professionals, underpinned by the perception of dementia as an older person's condition.

Studies described how these assumptions contributed to confusion and misdiagnosis, as healthcare professionals did not initially recognise dementia in younger individuals [4, 34, 37, 43]. Participants described a lack of specialised memory services tailored to YOD specifically ([33]; Lockeridge & Simpson, 2012). Caregivers and people with YOD reported being redirected between services or declined care due to their age, reflecting underlying age‐ based assumptions about who dementia services are tailored and intended for [4].

One doctor [family physician] said that he provides services only to elderly persons and asked us to turn to a geriatrician or psychiatrist, as they are the only ones who know the effect of the medicine on the brain in dementia. Then, I turned to a list of doctors, but no one agreed to meet him. They said they don't treat young patients.

(Female caregiver [4])

Confusion and a lack of awareness amongst health and social care professionals hindered access to appropriate workplace and healthcare support [37, 43]. Healthcare professionals reported that these issues were rooted in wider system and policy level factors, described as forms of *‘structural stigma’* [33].

There are systemic issues around ageism and then you translate that into the context of dementia and there are real system limitations and challenges that relate to a societal discomfort with dementia, that results in degrees of stigmatisation that impact on the way that policy work and systems work, then health systems… then we put the younger person into that context, what it does is it exacerbates and complicates all of those issues.

(Psychiatrist [33])

Services were seen as being part of a fractured health system with a lack of integration, leading to limited funding and accessibility for families [33]. This fragmentation was also reflected in the resources available, which were often out of date or inaccurate and did not align with the needs of people with YOD. As a result, participants described feeling that they did not ‘fit’ into services offered, leaving them feeling excluded, marginalised and frustrated [38].

Over time, his [husband with YOD] condition worsened because his formal caregiver did not know how to treat a young person with dementia; he [the professional caregiver] did not know how to engage him in daily activities, and they did not communicate for about 6 months. We had to switch registered caregivers several times.

(Wife [4])

Participants described experiences of being dismissed or deemed ineligible for support due to assumptions about their level of functioning, which contributed to feelings or marginalisation and discrimination [37]. In some cases, people with YOD were denied access to care packages and statutory payments because they did not fit expected profiles of need associated with dementia [38, 43].

… We asked for a care package so the care manager came out and she didn't believe it… she just said you didn't need any help so there's nothing I can do for you, because I was talking and everything else.

(Male living with YOD [37])

Several carers and people with YOD described difficulties accessing accurate and consistent information regarding entitlements to support, which contributed to experiences of exclusion [4, 34, 37, 38, 43]. Participants interpreted these experiences as a lack of recognition of YOD within services, reinforcing perceptions of being overlooked and unsupported in general [4].

My son checked every option available to try to get the funds we need to take care of him. Unfortunately, we are not eligible, so we didn't get any benefits.

(Wife [4])

Overall, these findings highlight how stigma can be embedded within healthcare systems and service structures, contributing to inequitable access to diagnosis, care and support for people living with YOD and their families.

#### 3.3.3 The Role of Stigma in Social Isolation

A third theme within this review illustrates how stigma associated with dementia resulted in people with YOD and their family members experiencing an increase in social isolation [4, 32, 35, 36, 37, 38, 40, 42, 46].

Participants with YOD and their caregivers reported that they felt different in society and ignored by peers, friends and professionals following the diagnosis and disclosure of YOD [32, 35, 36, 37, 46]. Participants with YOD felt they were on the peripheral of society and struggled to reconnect with others in their community due to stigma surrounding the diagnosis. Participants described feeling that people were physically distancing themselves from them [32, 36]. The reason for this was unclear, however other studies would suggest that people in society may fear that they will get the condition too, or they do not feel able to interact with a person who experiences cognitive difficulties [4, 35, 36].

A person with a memory disease today, they are thrown in the trash can right away.

(Female living with YOD [36])

She said that people, and to this day, people in her own village, will cross the road when they see her.

(Female caregiver [32])

Carers in two studies described experiencing courtesy stigma leading to them disconnecting from their social networks due to their increased caring responsibilities and sensing the negative attitudes from others extending to them [4, 37].

His family [her husband's siblings] don't accept it [the illness] and don't want to be part of it. They cut off contact with him.

(Wife [4])

I feel quite excluded and as by association, my husband feels the same.

(Female living with YOD [37])

As symptoms of YOD became more prominent, people with dementia and caregivers reported experiencing changes in their social and family roles. People with YOD described difficulties in communication and remembering information. The stigma associated with this change in social status, and the presenting symptoms, influenced the breakdown of social connections throughout these studies [4, 35, 37, 42].

Only one friend visited him; the rest disappeared. It is not pleasant – seeing him in his state. He was a communicative person, and now it is impossible to communicate with him; he does not understand what others say.

(Wife [4])

Participants reported feeling embarrassed during public outings and actively avoiding social situations and activities to protect the dignity of the person with dementia and reduce negative or uncomfortable experiences with the public (Blake & Hopper, 2011 [35, 37, 38, 40]).

And it got a bit embarrassing when you went out. You didn't want to. I knew people were looking at us but we'd sit somewhere out of view because he got so that he couldn't use a knife and fork properly, or he'd open a pat of butter and instead of putting it on his bread, he would eat it. Things like that, he never would have done before.

(Female caregiver [38])

Participants deemed wider networks as unable to understand their needs and they feared the stigma and prejudice attitudes that may come from disclosing the diagnosis. Participants reiterated that this withdrawal and active social isolation increased as the disease progressed.

#### 3.3.4 Overcoming Stigma

Several studies reported on the strategies that could be implemented on an individual and service level to help overcome the stigma experienced by the YOD population ([33, 34, 36]; Johannessen et al., 2024 [37, 42, 43, 44]).

Participants with YOD reported that they would attempt to redefine their dementia identities by challenging the misconceptions and stigma around the diagnosis through self‐advocacy online and representing living with dementia in a more positive way [33, 37, 44]. These studies outlined how providing education and knowledge about YOD within communities would increase access to diagnostic services [44]. Participants in one study used online platforms such as twitter to raise awareness about the lack of age appropriate, specialist services and campaign for change [44]. Participants described the benefits of becoming dementia activists and increasing the representation of YOD within society. This allowed participants to address negative and derogatory comments online and directly challenge the misconceptions about dementia in younger adults [37].

The key thing that would probably lead to the greatest number of increased [YOD] diagnoses would be community education. You know, if the community's knowledge about YOD is raised by 10% that is likely to result in a significant number of younger people presenting to diagnostic services for an answer. The same amount of money put into an existing tertiary diagnostic service is not going to achieve the same outcome, in terms of the number of new referrals.

(Psychiatrist [33])

I do tell people who I don't think will understand, because I think they need to learn a bit more about it… now I would step in and say, ‘Hang on a minute, that's not appropriate’.

(Female living with YOD [37])

The review findings suggest that several people with YOD try to find the confidence to disclose their diagnosis to family and friends and focus on the positive reactions received from those in society. This helped to combat the shame and self‐stigma that they were experiencing and allowed participants to be more comfortable and open about their diagnosis [37, 43].

… I realised that there was no reason for me to keep quiet about it, it's just an illness like any other illness, nothing to be ashamed of or anything like that, and I just was quite open about it, the way you would be open about anything else.

(Female living with YOD [37])

People with YOD in one study reported that sharing their diagnosis with employers, instead of concealing their symptoms, helped to foster understanding and empathy within workplace environments and was seen as crucial in facilitating adjustments in work and improving communication with employers [43]. In another study, when participants stayed *‘connected to the world’* around them and actively engaged in activities that continued to give them a sense of purpose and meaning, it helped them to overcome feelings of hopelessness [42]. To facilitate engagement in activities, several authors discussed the importance of peer support and age‐appropriate YOD services within dementia care pathways ([34, 36]; Johannessen et al., 2024 [37]).

Yes, you feel valued [when you keep working]. You feel that you matter. Then, you think, ‘hey, luckily, I still belong somewhere.’ That's the feeling that is really nice for me.

(Female living with YOD [43])

If you meet somebody who's newly diagnosed with it, or something like it, then you're helping them to learn to live with it.

(Female living with YOD [37])

When participants were offered the opportunity to speak with others who had YOD, it reduced the anxiety and shame they felt and helped foster more acceptance and positive perspectives for those impacted [34, 36]. These strategies helped reshape wider discourses around dementia, reduce public stigma and fear around the diagnosis.

## 4 Discussion

This qualitative systematic review synthesised evidence on how stigma is experienced by people with YOD and their families, shaping help‐seeking behaviours, diagnostic journeys, and access to support. To our knowledge, it is the first review to consolidate qualitative accounts of the experience of stigma across three participant groups including people with YOD, caregivers, and professionals globally.

A key finding of this review is that there is a persistent gap in public awareness about YOD. The lack of knowledge fosters misconceptions and negative assumptions about a YOD diagnosis, intensifying the fear and discomfort around the condition. Public perception and beliefs about YOD exacerbate feelings of shame, embarrassment and rejection for those impacted. This review emphasises the critical role of stigma in hindering and disrupting help seeking behaviours and acceptance of YOD diagnosis. The findings support previous dementia research that reported a loss of purpose, a decrease in self‐esteem and self‐efficacy experienced by people living with dementia at any age [20, 47]. Similarly, a systematic review by Nguyen and Li [48] reported limited general knowledge and prejudice from the public towards people with dementia at any age. People with dementia at all ages and types and their caregivers perceived discrimination from the public and this delayed help seeking behaviours, increased fear of disclosure and negative self‐images [48, 49]. In line with Goffman's modern conceptualisation of stigma, people appeared to see those with YOD and their carers as tainted by society and disqualified them from social acceptance because of the diagnosis [5].

While stigma is experienced in later onset dementia communities, the findings in the review suggest that it is intensified for YOD populations because they contend with limited public understanding and negative cultural beliefs about having dementia at a younger age. In line with Link and Phelan's [6] conceptualisations of stigma, this review found that people with YOD feel labelled by their diagnosis and ‘othered’ within society resulting in emotional distress, loss of identity and status. The review findings also mirror earlier conceptual frameworks of stigma coined by Erving Goffman in which an individual experiences a disqualification from social acceptance due to an attribute or characteristic, in this case YOD [3]. The lack of public acceptance propels people with YOD to ignore and mask their symptoms, as opposed to adapting and seeking support [50].

In this review, stigma was consistently associated with service level barriers to accessing adequate care. Stigma contributed to diagnostic delays due to limited YOD knowledge and confusion among professionals. Several studies describe the experiences of misdiagnosis, and a scarcity of age‐appropriate services pre and post diagnosis within the YOD diagnostic journey. Existing research has reported that people with YOD have different needs compared to older adults with dementia, and the provision of age‐appropriate services has been outlined as an essential component in the treatment pathway for YOD [51, 52]. Despite these recommendations, the lack of YOD specialist services and skilled healthcare professionals has been reiterated across the YOD literature [53, 54]. Furthermore, when people with YOD receive a diagnosis, they cannot access services that meet their unique psychosocial needs [17, 55] with care and support post diagnosis, often inadequate or unavailable [56, 57].

Research has shown that loneliness and social isolation impact on the physical health and psychological wellbeing of older adults and is a risk factor for developing dementia and other cognitive impairments [58, 59]. In this review, social isolation emerged as a prominent consequence of stigma for people with YOD and their caregivers. Participants withdrew from social networks and valued activities due to stigmatic experiences. An important finding of this review is understanding that stigma is a significant contributor to social isolation among people with YOD. Families are anticipating embarrassment and social exclusion, so they withdraw to minimise discomfort and shame. The concept of anticipated stigma and the self‐protective withdrawal from others plays a critical role in reducing help seeking behaviours for our YOD communities. Other published reviews on YOD found social isolation, withdrawal from social networks and feelings of embarrassment and loss of social status are widely reported by caregivers of YOD [60, 61]. This is consistent with findings from the Angela Project which showed that maintaining social networks was a fundamental need for families and people living with YOD and further supports the importance of relationship centred, post diagnostic support in mitigating isolation [62, 63].

This review builds on existing YOD research by highlighting a trajectory of inequalities that are experienced by this population due to stigma at an individual, community and societal level, in line with the Dementia Inequalities Model [64]. The literature around YOD and employment highlights the importance of flexible and individualised workplace adjustments to enable people to continue in their job roles following a diagnosis (Ritchie et al., 2025). This review showcases that even when people with YOD actively confront public stigma and seek support, they still encounter inadequate workplace adjustments, service designs that do not fit younger people's life stages, and limited financial support [49, 65]. These findings support existing research that illustrates how care remains misaligned with the needs of younger people living with dementia following a diagnosis [17, 55].

Clinical implications from this review include recommendations for enhanced education and specialist training on YOD for healthcare professionals in primary and secondary services to minimise confusion, misdiagnosis, and inadequate investigations. An increase in education will enable timely diagnoses and reduce patient distress, as outlined in previous research recommendations [66]. Given the current funding and staffing constraints within healthcare services nationally, raising awareness among all healthcare providers is critical to ensure appropriate referrals are processed effectively and individuals with YOD are not falling through service gaps. In previous studies exploring older age dementia populations, it was reported that education can enhance the service provision and reduce the stigma experienced by people living with the disease, which may be similar for the YOD population [39, 67, 68]. Clinical psychologists and other healthcare clinicians within dementia services who provide pre and post diagnostic support to the YOD community may be able to support and implement individual and system level strategies to help reduce the culture and practice of stigma within services. Encouragingly, professionals in one included study expressed their willingness to pursue this additional training on YOD [4].

Other recommendations for overcoming stigma include redefining dementia, improving the public perception of dementia, reducing anticipated stigma, and empowering individuals with YOD through identity affirming, age‐appropriate support. Individual strategies demonstrated in this review include supporting people to engage in new activities, and finding new purposes in life, such as voluntary roles and self‐advocacy. These findings appear consistent with research exploring living with dementia at all ages, and the importance of preserving hope and empowering those with dementia to advocate for their needs, to maintain wellbeing and reduce the negative consequences of stigma [69].

The National Dementia Strategy [70] and the Prime Minister's Challenge on Dementia (2020) emphasised the importance of early diagnosis, compassionate care and public awareness campaigns [71]. Many of the recommendations for overcoming stigma identified in this review rely heavily on local initiatives and voluntary sector leadership. Community level approaches, including the Dementia Friendly Communities and the Dementia Champions led by the Alzheimer's Society, have demonstrated effectiveness in reducing stigma and promoting inclusion. The findings of this review recommend that future public health awareness campaigns and empowerment efforts are operationalized within local commissioning and funding bodies, with specific consideration of YOD. In the UK context, the Young Dementia Network play a vital role in raising awareness, sharing resources and connecting carers, people living with YOD and professionals to help reduce stigma at a national level [72]. Other international services such as Lorenzo House, a community‐ based, global virtual support organisation for families living with YOD provide safe spaces for people to connect, share experiences, and advocate for dementia justice at local and national levels (Lorenzo House, 2024). This model of inclusivity and diversity showcases how community initiatives can complement existing public health frameworks and policy around YOD care provisions. This approach aligns with priorities outlined in the NHS Long Term Plan, which calls for tackling inequalities and improving dementia care pathways for all ages and dementia types [73]. By embedding YOD specific interventions into these established frameworks, policymakers and professionals can move beyond tokenistic goals toward practical, evidence‐based action to reduce the experience of stigma.

### 4.1 Strengths and Limitations

This review employed a rigorous synthesis approach with additional reviewers contributing to both stages of the screening process, as well as the quality assessment, data extraction and analysis of included studies. This review was conducted robustly, with searches across five databases, and searches of included papers' reference lists. This method ensured the evidence synthesised was representative of the available literature on the review topic. The studies included were conducted across twelve countries, offering a broad international perspective in this review. The inclusion of qualitative research provided rich, nuanced insights into the lived experiences of three participants groups enhancing the depth and validity of the findings. Furthermore, this review represents one of the first attempts to synthesise evidence specifically on stigma in YOD, addressing a critical gap in the literature and offering a foundation for future policy and practice recommendations within dementia care.

Several limitations of this review also warrant consideration. The diversity of the included studies sample was difficult to ascertain because participant's ethnicity was not reported in eleven studies. Furthermore, diagnostic timelines and service uptake among ethnic minority groups were rarely reported within studies. This restricts our understanding of socio‐cultural variations in the experience of stigma and may bias findings toward majority or White populations [74]. The CASP quality assessment tool revealed weaknesses in reflexivity across the included studies. Thirteen studies lacked sufficient consideration of the researcher participant relationship and the influence of positionality, subjectivity, and potential bias on data collection and analysis. The absence of reflexivity may affect the credibility and trustworthiness of the evidence base being analysed [75]. The search strategy was limited to peer‐reviewed articles published in English and German, which introduces language and cultural bias and may exclude valuable insights from other regions, thereby reducing the generalisability of the findings [48]. Lastly, although all the included studies explored the lived experiences of YOD population, only one of the included studies aimed to examine the experience of stigma specifically [4]. This may narrow the scope of evidence of this review and limit the strength of the conclusions drawn.

While the review offers a robust synthesis and makes a novel contribution to our understanding of stigma in YOD, the methodological and contextual limitations outlined by this review highlight recommendations for future research. Future research could adopt more inclusive sampling, strengthen reflexivity practices in qualitative designs and ensure transparent reporting of demographic characteristics. There is a lack of evidence on identifying the anti‑stigma strategies that work best for YOD populations. Future research could examine the effectiveness of these individual and service level strategies using quantitative and qualitative methods to inform national dementia policy and provision [39]. It is important that perspectives from ethnic minority groups are included in any future research to identify difference or additional barriers and facilitators to timely diagnosis and support for these communities.

## 5 Conclusion

To our knowledge, this systematic review is the first to synthesise qualitative literature on the experiences of stigma in YOD globally. This review highlights the pervasive and multifaceted nature of YOD, offering insights into individual and service level strategies needed to mitigate the impact of stigma. The findings of this review show the urgent need for age‐appropriate support services alongside public and professional education on YOD to address misconceptions about dementia in younger adults. Community and government‐led initiatives can help dismantle negative stereotypes, reduce social isolation and improve access to early diagnosis and tailored support for our YOD communities.

## Search Strategies for Each Database

**Web of Science**

((‘young onset dementia’ OR ‘early onset dementia’ OR ‘presenile dementia’ OR ‘younger onset dementia’) AND (Stigma* OR Discriminat* OR Prejudic* OR Marginal* OR Bias* OR Attitude* OR Exclu*))

**APA PsycINFO**

((young onset dementia) OR (early onset dementia) OR (presenile dementia) OR (younger onset dementia)) AND ((Stigma*) OR (Discriminat*) OR (Prejudic*) OR (Marginal*) OR (Bias*) OR (Attitude*) OR (Exclu*))

**Medline**

((‘young onset dementia’ or ‘early onset dementia’ or ‘presenile dementia’ or ‘younger onset dementia’) and (Stigma* or Discriminat* or Prejudic* or Marginal* or Bias* or Attitude* or Exclu*))

**Scopus**

((‘young onset dementia’) OR (‘early onset dementia’) OR (‘presenile dementia’) OR (‘younger onset dementia’)) AND ((Stigma*) OR (Discriminat*) OR (Prejudic*) OR (Marginal*) OR (Attitude*) OR (Exclu*))

**Pubmed**

((‘young onset dementia’) OR (‘early onset dementia’) OR (‘presenile dementia’) OR (‘younger onset dementia’)) AND ((Stigma*) OR (Discriminat*) OR (Prejudic*) OR (Marginal*) OR (Attitude*) OR (Exclu*))

## Author Contributions

**Fiona Lowe:** conceptualisation, methodology, data curation, writing – original draft, formal analysis. **Kerry Hanna:** conceptualisation, methodology, formal analysis, writing – review and editing, supervision. **Sarah Evans:** data curation. **Naheed Tahir:** validation. **Clarissa Giebel:** conceptualisation, methodology, supervision, writing – review and editing, validation, formal analysis.

## Ethics Statement

The authors have nothing to report.

## Conflicts of Interest

The authors declare no conflicts of interest.

## Data Availability

Data sharing not applicable to this article as no datasets were generated or analysed during the current study.
